# Enhancement of *TRP* Gene Expression and UV Absorption by Bioconverted Chestnut Inner Shell Extracts Using *Lactiplantibacillus plantarum*

**DOI:** 10.3390/molecules27154940

**Published:** 2022-08-03

**Authors:** So-Hee Kim, Suh-Hee Yoem, Jun-Hee Kim, Ji-Woo Hong, Ye-Sol Oh, Jin-Woo Kim

**Affiliations:** 1 Department of Food Science, Sun Moon University, Natural Science 118, 70 Sunmoon-ro 221, Tangjeong-myeon, Asan-si 336-708, Korea; ssohiii@naver.com (S.-H.K.); yshee0017@naver.com (S.-H.Y.); jun981014@naver.com (J.-H.K.); hgw3130@naver.com (J.-W.H.); dhtpws0000@naver.com (Y.-S.O.); 2 Next-Generation Semiconductor Technology Center, Sun Moon University, 70 Sunmoon-ro 221, Tangjeong-myeon, Asan-si 336-708, Korea; 3 FlexPro Biotechnology, Natural Science 128, 70 Sunmoon-ro 221, Tangjeong-myeon, Asan-si 336-708, Korea

**Keywords:** bioconversion, *Lactiplantibacillus plantarum*, chestnut inner shell, *TRP-1*, *TRP-2*, UV absorption

## Abstract

In this work, the suppression of tyrosinase-related genes, including an improvement in UV absorption effects of bioconverted CS extracts (BCS), was investigated to improve the skin-whitening effect. Total polyphenols and total flavonoids, which are bioactive components, increased 2.6- and 5.4-times in bioconversion using *Lactiplantibacillus plantarum* SM4, respectively, as compared to ultrasound-assisted extracts (UCS). The effect of BCS on radical scavenging activity, UV-A absorption, and tyrosinase activity inhibition, contributing to skin-whitening, were 1.3-, 1.2-, and 1.2-times higher than those of UCS, respectively. The main component identified in high-performance liquid chromatography (HPLC) was gallic acid in both UCS and BCS, which increased by 2.9-times following bioconversion. The gene expression of tyrosinase-related proteins, including *TRP-1* and *TRP-2* genes, was studied to confirm the suppression of melanin synthesis by BCS in order to identify the skin-whitening mechanism, and BCS decreased both genes’ expression by 1.7- and 1.6-times, demonstrating that BCS effectively suppressed melanin synthesis. These findings imply that the chestnut inner shell can be employed as a cosmetic material by simultaneously inhibiting melanogenesis and enhancing UV-A absorption through bioconversion using *L. plantarum* SM4.

## 1. Introduction

As a result of rapid industrialization, the amount of industrial by-products and greenhouse gases, such as carbon dioxide, methane, nitrous oxide, hydrofluorocarbon, perfluorocarbon, and sulfur hexafluoride, has increased considerably, and the depletion rate of Earth’s protective ozone layer appears to have accelerated over the past few years [1]. Continuous monitoring of air quality reveals the depletion of the ozone layer accelerates exposure of UV to the skin and a 10% loss in the ozone layer increases the UV reaching the Earth’s surface by 1% [2]. The UV region covers the wavelength range of 100–400 nm and is classified into three bands, including UV-A (320–400 nm), UV-B (290–320 nm), and UV-C (200–290 nm) [3]. As sunlight passes through the atmosphere, most UV-B and UV-C are absorbed by the ozone, water vapor, oxygen, and carbon dioxide, whereas the majority of UV-A reaches the Earth’s surface unabsorbed. Therefore, most of the UV rays that reach the skin are UV-A, which is absorbed into the skin epidermis and rapidly stimulates the generation of reactive oxygen species (ROS), such as hydrogen peroxide (H_2_O_2_), superoxide radicals (O_2_^−^), hydroxyl radical, nitric oxide (NO), and peroxynitrite (ONOO^−^) [4].

ROS oxidize unsaturated fatty acids, causing lipid peroxidation. Lipid peroxidation plays a critical role in apoptosis, autophagy, and ferroptosis via damage to DNA, RNA, enzymes, proteins, and cell membranes, so it is imperative to develop UV-A-protective cosmetics [5]. The extracellular matrix (ECM) is known as an extracellular connective tissue matrix with collagen and non-collagen components in complicated aggregates, with a highly dynamic and diverse structure that plays multiple roles in living organisms [6]. Its integrity and homeostasis are essential for normal tissue development and organ physiology, as well as offering unique properties of skin protection, moisture retention, elasticity, tensile strength, and compressibility [7]. UV-induced ROS enhance the activity of matrix metalloproteinases (MMPs), including collagenases, gelatinases, stromelysins, matrilysins, and membrane-type MMPs, which degrade ECM’s collagen, elastin, and glycoproteins, resulting in skin aging through reduced elasticity [8]. Furthermore, ROS activate tyrosinase-related protein-1 (*TRP-1*) and tyrosinase-related protein-2 (*TRP-2*) genes, which are two major transcriptional genes involved in the melanin synthesis and regulation of the quality of melanin [9]. *TRP-2* converts DOPA–chrome to 5,6-dihydroxyindole-2-carboxylic acid (DHICA), while *TRP-1* oxidizes DHICA to a carboxylated indole-quinone, which is then converted to melanin [10]. Therefore, synthetic antioxidants, including butylated hydroxytoluene, butylated hydroxyanisole, tertiary butylhydroquinone, and propyl gallate, have been widely used in the cosmetics industry to suppress melanin synthesis and wrinkle generation caused by ROS [11]. On the other hand, natural antioxidants have been extensively studied to replace synthetic antioxidants because they have the disadvantage of cytotoxicity, liver damage, hepatocellular carcinoma, and carcinogenicity, known as shortcomings of synthetic antioxidants [12]. Currently, for the commercialization of natural antioxidants, numerous studies are being conducted to overcome the shortcomings of natural antioxidants by increasing natural antioxidants’ ROS scavenging potential, price competitiveness, and stability.

Bioconversion, also known as biotransformation, is a technology that converts macro-organic components in plant or animal biomass to valuable products or energy sources by biological processes, such as specific microorganisms or enzymes [13]. Over the past few decades, bioconversion has improved its competitiveness due to the rapid development of fermentation, metabolic engineering, and enzyme engineering, and is highlighted as a next-generation technology because it successfully converts macromolecules to produce various bioactive components, facilitates bioavailability, and supports commercial-scale production [14].

Chestnut (*Castanea sativa*) inner shell (CS) is a by-product that accounts for 50% of the total output of chestnut total production but is wasted because it is unsuitable for food or livestock feed [15]. While research on CS has not been actively conducted, Braga et al. reported substantial amounts of polyphenols, such as quercetin, ellagic acid, naringenin, gallic acid, and tannin, in CS, suggesting that bioactive ingredients with added value can be obtained from the chestnut shell through a more effective extraction method [16]. The purpose of this study is to improve skin-whitening and UV-A absorption component production by performing bioconversion of CS using *Lactiplantibacillus plantarum* SM4. Therefore, the intracellular β-glucosidase was recovered after maximizing β-glucosidase production at 37 °C, the optimal growth temperature of *L. plantarum* SM4. Then, bioconversion was performed at 45 °C to maximize the activity of β-glucosidase. After that, in order to confirm the possibility of bioconverted CS extracts (BCS) being used as a high-value-added cosmetic material, high-performance liquid chromatography (HPLC) analysis was conducted to identify the main components of BCS and ultrasound-assisted extracts (UCS), and the skin-whitening and UV-A absorption mechanisms were evaluated to determine the potential of CS for use as high-value cosmetic materials.

## 2. Results and Discussion

### 2.1. Measurement of Antioxidant Activity

**Figure 1:**
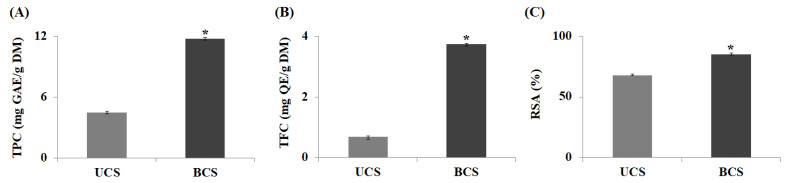
Comparisons of TPC (**A**), TFC (**B**), and RSA (**C**) changes in UCS and BCS by bioconversion. UCS: ultrasound-assisted extracts of chestnut inner shell; BCS: bioconverted chestnut inner shell extracts; TPC: total polyphenol content; TFC: total flavonoid content; RSA: radical scavenging activity. The statistical analysis of the data was carried out by use of a *t*-test. Values are statistically significant compared to UCS at * *p* < 0.05.

By comparing the total polyphenol content (TPC) and total flavonoid content (TFC) of UCS and BCS, it was confirmed that the production of bioactive components increased through bioconversion. The TPC of UCS and BCS was 4.5 ± 0.13 and 11.8 ± 0.11 mg GAE/g DM, which increased 2.6-times by bioconversion. Similarly, the TFC of UCS and BCS increased by 5.4-times from 0.69 ± 0.02 to 3.75 ± 0.07 mg QE/g DM after bioconversion (Figure 1A, B). The difference in the increase rate between polyphenol and flavonoid was that the phenyl group produced by hydrolysis of the polyphenol in bioconversion combined with the flavan C_6_-C_3_-C_6_ skeleton in flavonoid synthesis to increase TFC yields, resulting in a greater increase rate in TFC compared to TPC [17]. ROS are oxygen-containing radicals that may exist independently in one or more unpaired electrons and are removed by obtaining electrons or hydrogen from antioxidants, such as polyphenols. The radical scavenging activity (RSA), indicators of antioxidant potential, of UCS and BCS were measured at 68.2 ± 0.88% and 85.4 ± 0.61% respectively, increasing 1.3-times through bioconversion (Figure 1C). This is consistent with a study showing that bioconversion using lactic acid bacteria from bamboo shoots enhanced RSA by 17.7% and it was proven that bioconversion is an effective tool that could increase the antioxidant potential in a variety of plants [18].

Polyphenols are a type of aromatic alcohol that is a large family, naturally found in plants, which are characterized by multiple phenol units with one or more hydroxyl groups. In many cases, they exist in polymers or the forms of ester binding with macromolecules, including proteins, polysaccharides, and lignin, in the plant cell wall [19]. In addition, they are widely known as secondary metabolites, with high antioxidant activity through hydrogenation by a hydroxyl group and resonance stabilization of the phenolic ring structure [20]. There are around 8000 different forms of polyphenols, which are divided into four categories: flavonoids, phenolic acids, polyphenolic amides, and additional polyphenols, such as resveratrol, ellagic acid, curcumin, and lignan [21]. Among them, flavonoids, including aglycone, glycosides, and methylated derivatives, are polyphenols that color fruits and vegetables, which are beneficial to humans and are the most abundant in plants [22]. As with most polyphenolic antioxidants, the number and position of the B-ring hydroxyl groups in flavonoids have a significant impact on the mechanisms of antioxidant action. A 3,4-catechol structure in the B-ring greatly enhances antioxidant activity [23]. Therefore, in plants, flavonoids are known to play an important role in ROS scavenging, UV protection, symbiotic nitrogen fixation, and floral coloring. They also exhibit antioxidant activity by donating hydrogen atoms from their multiple hydroxyl groups to ROS as well as inhibiting the production of ROS by intracellular lipoxygenase and lowering the rate of ROS production.

Our results reaffirmed that bioconversion had a significant effect on the increase in RSA as well as TPC and TFC. Polyphenols in plants are a representative antioxidant that stabilize ROS to prevent cell oxidation, mutation, and aging, and the rise in RSA observed in this study was expected to be related to the increase in polyphenols and flavonoids in extracts due to bioconversion.

### 2.2. Measurement of Tyrosinase Activity Inhibition and UV-A Absorption

**Figure 2:**
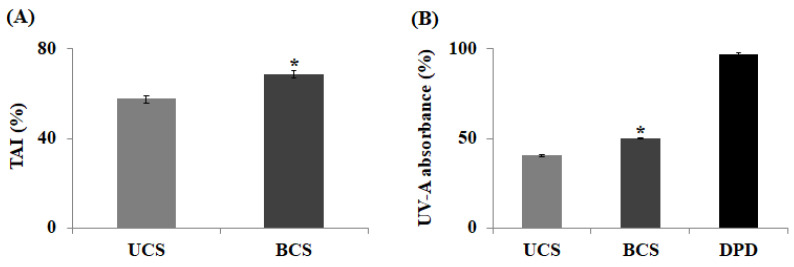
The values of TAI change (**A**) and UV-A absorption change (**B**) by bioconversion using *L. plantarum* SM4. UCS: ultrasound-assisted extracts of chestnut inner shell; BCS: bioconverted chestnut inner shell extracts; TAI: tyrosinase activity inhibition. All values are means in triplicate measurement and the statistical analysis of the data was conducted by a *t*-test. Bars marked with asterisks are significantly different from the control (* *p* < 0.05).

Tyrosinase is an enzyme that initiates the synthesis of melanin, in which tyrosine is converted to melanin after several intermediate phases of DOPA, DOPA–quinone, and DOPA–chrome via a series of auto-oxidation and enzymatic reactions. Therefore, to avoid melanin formation and enhance skin-whitening, the activity of the major enzyme in melanin pigment formation, tyrosinase, must be inhibited. To this end, when comparing tyrosinase activity inhibition (TAI) of UCS and BCS to confirm the effect of enhancing skin-whitening by bioconversion, TAI of BCS was measured at 68.8 ± 0.38%, an increase of 1.2-times compared to UCS (Figure 2A). These findings outperform previous studies that a 1.2-times increase in TAI to 60.6%, through the bioconversion of *Sophhorae fructus*, confirming BCS effectively inhibits the tyrosinase activity [24]. Therefore, BCS can be used as a natural material for the skin-whitening effect because it has an excellent effect in inhibiting melanin production compared to other plant extracts.

Furthermore, UV induces ROS accumulation, DNA damage, oxidation of lipids, and activation of MMPs in the skin, such as fibroblasts, keratinocytes, macrophages, endothelial cells, and eosinophils. This damage causes a decrease in collagen and elastic fibers in the dermis, leading to photoaging. In addition, chronic UV-A exposure induces a variety of skin disorders and skin cancers via complex pathways mediated by UV-induced ROS [25]. Therefore, to prevent photoaging caused by UV-A, it is essential to discover natural components that effectively absorb UV-A. As shown in Figure 2B, the UV-A absorption rate for UCS was 40.3 ± 0.05%, while that for BCS was 50.0 ± 0.06%, showing an increase of 1.2-times via bioconversion. The increase in UV-A absorption rate is most likely due to the conversion of UV-absorbing polyphenols bound to lignin, proteins, or polysaccharides in the cell wall into free polyphenols by enzyme hydrolysis and present in greater quantities in ferments. Although the absorption rate of BCS in this study was lower than 97.3% of DPD, it is expected that through extraction and enzyme conversion process optimization, the UV-A absorption rate can be improved, and it is estimated that a certain portion or all of the synthetic absorbents, DPD, can be replaced with natural UV-A absorbents, such as BCS.

### 2.3. Measurement of Cell Viability

**Figure 3:**
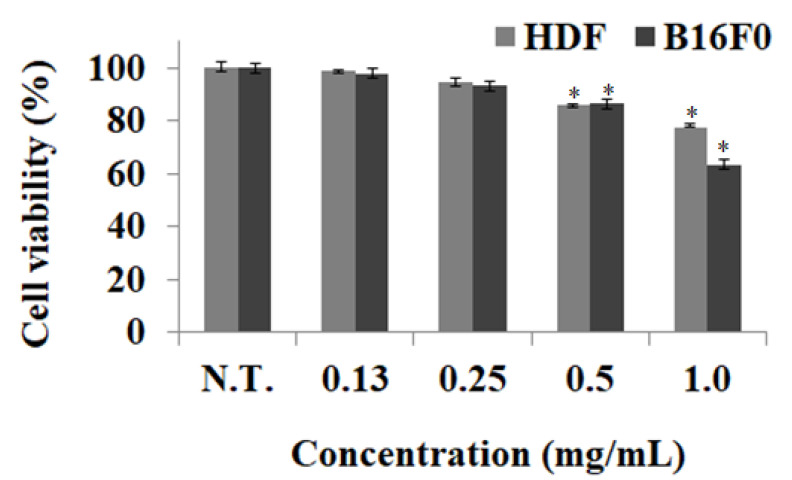
Inhibitory effects BCS on cell viability of HDF and B16F0. BCS: bioconverted chestnut inner shell extracts. The cell growth of the control group with the nontreated group (N.T.) at 24 h was represented as 100%. Results represent mean ± standard deviation (*n* = 3). The statistical analysis of the data was carried out by use of a *t*-test. Values are statistically significant at * *p* < 0.05 vs. respective control group.

Since cosmetics are used for long term on the skin, cytotoxicity experiments are important in securing the safety of cosmetic ingredients and protecting consumer health by providing skin safety information, along with data for functional evaluation. Cytotoxicity experiments were performed to investigate whether the BCS was toxic to skin cells and determine the concentration for safe treatment. When cell viability was measured after 0.0–1.0 mg/mL treatment of BCS in HDF and B16F0, cytotoxicity was not observed in both cells below 0.25 mg/mL and the BCS treatment was found to have a cell survival rate of more than 90% at concentrations of 0.0–0.25 mg/mL in both cells (Figure 3). Therefore, when developing cosmetic materials with skin-whitening and UV-A absorption effects using the BCS, cellular nontoxicity could be secured below 0.25 mg/mL of BCS. According to the result of Hridya’s study, it was confirmed that brazilein, a naturally occurring red dye obtained from *Paubrasilia echinata*, showed cell survival of 80.0% at the same treatment concentration, confirming that BCS treatment was safer compared to other natural materials [26].

### 2.4. Measurement of Gene Expression Levels

**Figure 4:**
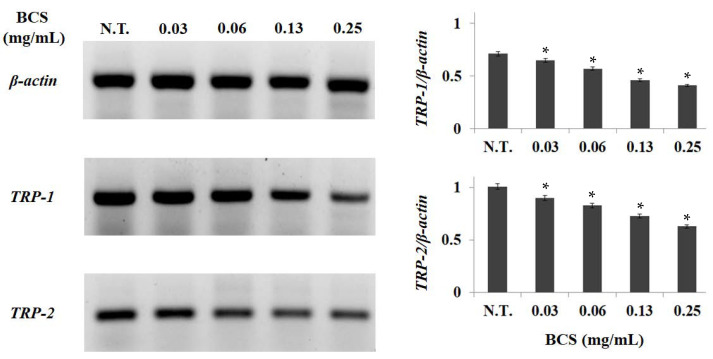
Effect of BCS on mRNA expressions levels of *TRP-1* and *TRP-2* in HDF cells. BCS: bioconverted chestnut inner shell extracts. The statistical analysis of the data was carried out by use of a *t*-test. Values are statistically significant compared to *β-actin* at * *p* < 0.05. *β-actin* was used as internal control.

Melanin is synthesized through an enzymatic cascade regulated by tyrosine, *TRP-1*, and *TRP-2*, and the inhibition of the activity of these major genes is known as an effective strategy to reduce melanin synthesis from tyrosine. To measure the effects of BCS on the inhibition of *TRP-1* and *TRP-2* expression, which promotes melanin synthesis, B16F0 was treated with BCS at different concentrations of 0.0–0.25 mg/mL and checked by RT-PCR. *TRP-1* and *TRP-2* expression decreased in concentration-dependent patterns in all test ranges (Figure 4).

The results of this study were similar to those of biorenovation-assisted modification of *Ligustrum japonicum* extract for a skin-whitening effect by Sim et al., where bioconverted extracts also showed a concentration-dependent decrease in *TRP-1* and *TRP-2* expression [27]. The promotion of melanin production can be controlled by tyrosinase, *TRP-1*, and *TRP-2*. Tyrosinase promotes a hydroxyl reaction to convert tyrosine to DOPA and DOPA is converted to DOPA–quinone. *TRP-1* oxidizes DHICA to carboxylated indole-quinone, and *TRP-2* acts as a DOPA–chrome automerase, resulting in a DOPA–chrome switch to DHICA. Thus, the activity inhibition of tyrosinase, *TRP-1*, and *TRP-2* plays an important role in inhibiting melanogenesis and enhancing skin-whitening.

In our previous studies, TPC, TFC, and RSA were measured to evaluate the effect of increasing antioxidant activity by BCS. As a result, TPC, TFC, and RSA were significantly increased in BCS compared to UCS after bioconversion, suggesting that the antioxidant potential of BCS, which inhibits melanin synthesis through the inhibition of ROS formation, is associated with the inhibition of *TRP-1* and *TRP-2* activity. Accordingly, it was confirmed that BCS has the effect of inhibiting melanin synthesis by suppressing *TRP-1* and *TRP-2* in B16F0, so it is highly likely to be used as a functional cosmetic related to whitening by scavenging ROS.

### 2.5. LC-MS/MS Analysis

**Figure 5:**
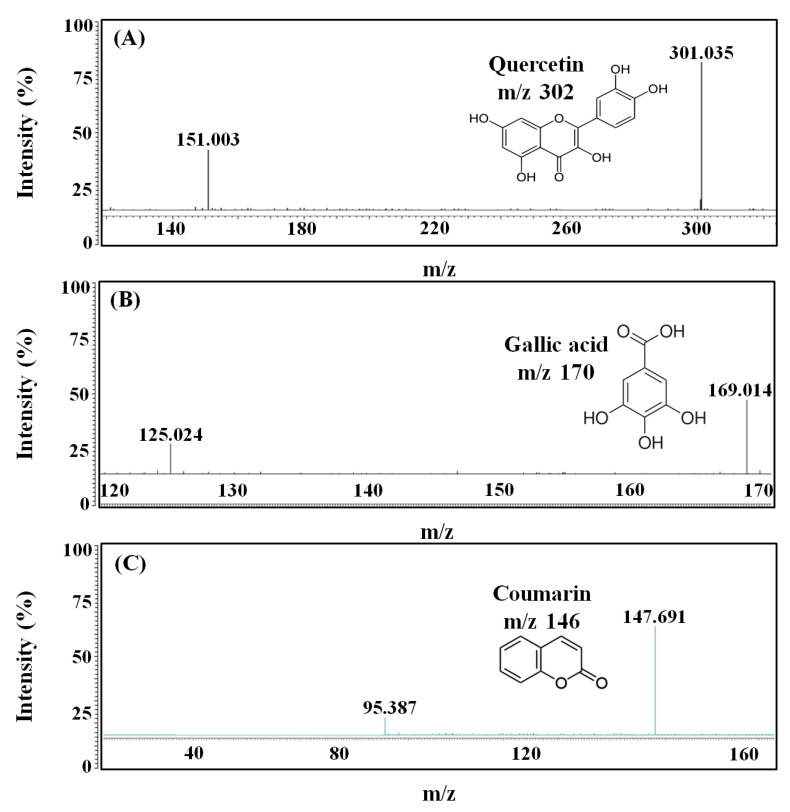

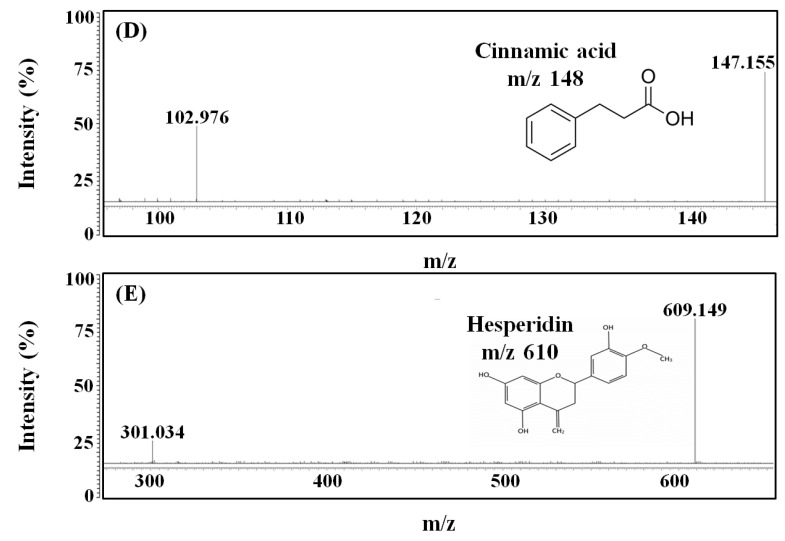
LC-MS/MS spectra for the analysis of main components in BCS. (**A**) quercetin, (**B**) gallic acid, (**C**) coumarin, (**D**) cinnamic acid, and (**E**) hesperidin. BCS: bioconverted chestnut inner shell extracts.

LC-MS/MS analysis was performed for the identification of components with bioactive properties, including antioxidant, skin-whitening, and UV-A absorption in BCS. As a result, five components, including gallic acid, coumarin, cinnamic acid, quercetin, and hesperidin, were identified by the presence of molecular ion peaks in form of MH- at *m*/*z* of 169.0, 147.0, 165.1, 301.0, and 609.2, respectively (Figure 5).

As previously stated, BCS contains components with varied bioactive properties, one of which is gallic acid, a phenolic acid found in several plants, including fruits, nuts, wine, and tea. It possesses antioxidant, antibacterial, anticancer, and antiobesity properties, as well as skin-whitening properties [28]. According to many studies, gallic acid has a skin-whitening effect by suppressing tyrosinase activity by lowering the production of MITF, which is required for melanin synthesis and transportation, as well as a decrease in the activity of DOPA–chrome tautomerase (Dct), implicated in melanin formation [29]. Coumarin is a heterocyclic component that has been linked to health benefits, such as the lower risk of cancer, diabetes, cardiovascular disease, and brain disease [30]. Several studies have shown that umbelliferone and esculetin, present in the ring of coumarin, strongly inhibit tyrosinase and act as effective skin-whitening agents. In particular, it is known that esculetin not only inhibits DOPA oxidation by competition with tyrosinase but also exhibits antioxidant activity by inhibiting oxidative polymerization of intermediate products derived from DOPA in melanocytes [31]. In addition, cinnamic acid, classified as a structural and functional component of plant cell walls, has dietary activity and is used as a preventive or therapeutic agent in a variety of oxidative-stress-related disorders, including cancer, atherosclerosis, and inflammatory damage [32]. Components with the excellent whitening effect of BCS include cinnamic acid and quercetin, which competitively inhibits the activity of diphenolase, involved in melanin synthesis that converts L-tyrosine to DOPA–quinone [33]. Quercetin is also a polyphenolic flavonoid that is common in plants and has a range of biological activities, such as antioxidant, anticancer, antiviral, and angiogenesis inhibitory properties [34]. Quercetin is also known to have skin-whitening effects, especially by acting on flavonols without 3-hydroxyl groups to inhibit the enzymatic oxidation of DOPA, thereby inhibiting melanin synthesis [35]. Finally, hesperidin, a bioflavonoid found in citrus, has been studied as a therapeutic component with antioxidant and anticancer effects [36]. It is found in a variety of plants and is known as one of the representative functions as a topical sunscreen by shielding phosphatidylcholine liposomes from UV peroxidation [37]. In addition, hesperidin acts as a strong anti-photoaging factor by inhibiting the expression of MMP-9 and regulating the signaling pathway of kinases, which are mitogen-activating proteins [38]. As a result, the skin-whitening and UV-A absorption effects of BCS observed in this study are thought to be derived from five main components identified by LC-MS/MS.

### 2.6. HPLC Analysis

**Figure 6:**
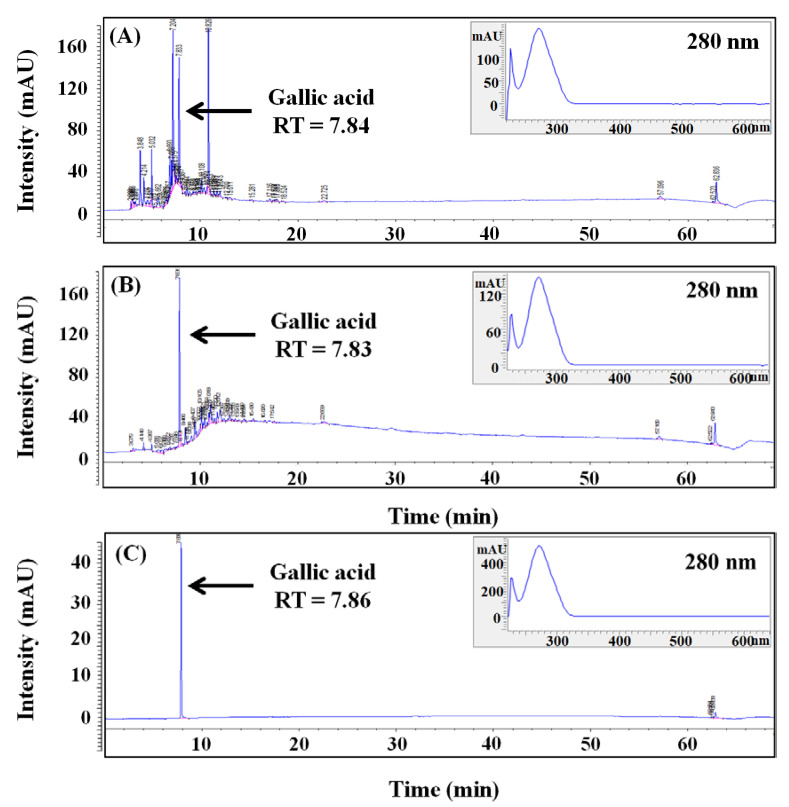
HPLC chromatograms for quantitative and qualitative analysis for the determination of gallic acid in UCS and BCS. Chromatogram and DAD spectrum (190–640 nm) of gallic acid from UCS (**A**) and BCS (**B**). Chromatogram and DAD spectrum (190–640 nm) of gallic acid standard (**C**, 0.1 mg/mL). UCS: ultrasound-assisted extracts of chestnut inner shell; BCS: bioconverted chestnut inner shell extracts.

Five main components in BCS were identified through the previous LC-MS/MS analysis. As a follow-up experiment, HPLC analysis was conducted on UCS and BCS to determine concentrations of each component and analyze the changes in components by bioconversion. For quantitative and qualitative examination of major components of UCS and BCS, the retention time (RT) of the main peaks and absorption spectra were compared and the RT of the main peaks was detected at 7.84 and 7.83 min, respectively (Figure 6). They are consistent with the RT of the gallic acid standard of 7.83 min, and the calculated concentrations based on the peak areas were 4.04 mg/g in UCS and 11.63 mg/g in BCS, indicating that the bioconversion resulted in an approximately 2.9-times increase in gallic acid. Gallic acid is a kind of tannin found in many plants, including green tea, coffee, black tea, and grape, and is known as a member of polyphenols with effective antioxidant, antibacterial, and anticancer activities.

The use of gallic acid as a functional material is increasing in the cosmetics industry. Recent studies have reported that gallic acid inhibits the activation of tyrosinase and scavenges ROS through an electron donation, thereby showing high antioxidant activities. The increase in gallic acid concentration after bioconversion is assumed to be caused by the hydrolysis of gallic acid bound to the plant cell matrix or present in the form of gallotannin into gallic acid by enzymes, such as β-glucosidase, decarboxylase, and reductase, produced by microorganisms. Therefore, in this study, among the main components identified by LC-MS/MS analysis, it was confirmed that the component with the highest concentration in UCS and BCS was identified as gallic acid. Then, the use of *L. plantarum* SM4 to bioconvert the bioactive component in UCS could contribute to improving secondary metabolite production while also increasing the utility and economic feasibility of agricultural by-products.

## 3. Materials and Methods

### 3.1. Materials and Reagents

Chestnuts were purchased from Utgolfarm (Seongnam, Korea) and the outer shell was peeled off to obtain CS. CS was dried using a forced convection drying oven (VS-1202D4N, Vision Bionex, Buchoen, Korea) at 60 °C for 48 h and then pulverized into a particle size less than 0.4 mm using a food grinder (HMF-3000S, Hanil, Buchoen, Korea). Folin–Ciocalteu, sodium carbonate, aluminum chloride, potassium acetate, 1-3,4-dihydroxyphenylalanine (L-DOPA), mushroom tyrosinase, 2,2-diphenyl-1-picrylhydrazyl (DPPH), gallic acid, quercetin, kojic acid, and ascorbic acid were obtained from Sigma-Aldrich (St Louis, MO, USA). MRS medium for *L. plantarum* culture was purchased from BD Difco (Sparks, MD, USA). Fetal bovine serum (FBS), Dulbecco’s modified eagle medium (DMEM), trypsin-EDTA, 3-(4,5-dimethylthiazol-2-yl)-2,5-diphenyltetrazolium bromide (MTT), and the dimethyl sulfoxide (DMSO) for cell culture and viability testing were purchased from Thermo Fisher (Waltham, MA, USA).

### 3.2. Ultrasound-Assisted Extraction

For the extraction of bioactive components from CS, 1.0 g of the dried sample was placed in a 15 mL pressure tube (Ace glass, Vineland, NJ) and mixed with 10.0 mL of 80% ethanol using a vortex mixer (VM-10, Daihan, Ltd., Wonju, Korea) for 1 min. Extraction was carried out at 70 °C and 60 min in the ultrasound extractor (250 W, SD-D250H, Daihan, Wonju, Korea). Then, the extract was centrifuged at 10,000 rpm for 10 min (236R, Labogene, Seoul, Korea) and filtered through a 0.22 µm PVDF syringe filter (PVDF2025A, Hyundai micro, Seoul, Korea) prior to HPLC analysis.

### 3.3. Isolation and Characterization of Microorganism

For microorganism selection, the solid medium was prepared with MRS containing 1.5% agar. Kimchi was collected from 21 regions in Korea and the broth was diluted 100-times with MRS medium before being spread on an MRS agar plate and cultured at 37 °C for 24 h. Strains were separated and cultured from 29 kimchi collected from the various provinces of Korea and 13 strains with high acidity during fermentation were selected and cultured on plate. Subsequently, 18 enzyme activities, including β-glucosidase, were compared using API ZYM kit (BioMerieux, Etoile, France) and strains with the highest β-glucosidase activity were selected. Selected colony was transferred to 10.0 mL of MRS medium and incubated at 37 °C for 48 h. Then, 18 different enzyme activities were tested using the analytical profile index (API) kit (BioMerieux, SA, Lyon, France) for the selection of the strain with the highest β-glucosidase activity. For the strain identification, DNA was extracted from the strain and the PCR (MJ Research, PTC 225, Watertown, MA, USA) was conducted using the 16S rRNA gene domain comparison. The 16S rRNA gene was amplified from chromosomal DNA using the universal bacterial primers 27F (50-AGA GTT TGA TCM TGG CTC AG-30) and 1492R (50-TAC GGY TAC CTT GTT ACG ACT T-30). The amplified PCR product was purified using the QIAquick PCR purification kit (Qiagen, Hilden, NRW, Germany) and the purified PCR product was sequenced using the Macrogen’s service (Seoul, Korea). The 16S rRNA gene sequence homology search was performed at the National Centre of Biotechnology Information (NCBI) database using BLASTN and the selected strain was named *L. plantarum* SM4.

### 3.4. Bioconversion

The production of enzymes for biotransformation was achieved by fermenting lactic acid bacteria, *L. plantarum* SM4. The MRS medium was distributed to the 250 mL Erlenmeyer flask and the initial pH was adjusted to 7.0 using 1 M HCl and 1 M NaOH. The medium was sterilized at 121 °C for 15 min using an autoclave (SAC05060P, Daihan, Gangwon-do, Korea) and placed on a clean bench for air cooling. After inoculating 1.0% of *L. plantarum* SM4 seeds in 50.0 mL of MRS medium, they were incubated and cultured at 37 °C, the optimal growth temperature of *L. plantarum*, 200 rpm for 24 h in a reciprocal shaking incubator (SI-87, U1Tech, Gyeonggi-do, Korea) for maximum enzyme production. Thereafter, in order to maximize the enzymatic hydrolysis efficiency, bioconversion was performed at 45 °C, the optimum activity temperature of β-glucosidase, for 48 h to maximize the enzyme activity.

For bioconversion, the extracts obtained from UCS and the fermentation medium were mixed at a ratio of 2:1 (*v*/*v*) and further fermented at 37 °C and 200 rpm for 48 h to produce maximum intracellular enzymes. After disruption of cells with a homogenizer (HG-15A, Daihan, Gangwon-do, Korea), for obtaining intracellular enzymes, the pH of the fermentation medium was adjusted to 5.0 ± 0.1 with 1 M HCI and 1 M NaOH, and bioconversion was further carried out at 45 °C for additional 48 h.

### 3.5. Measurement of Total Polyphenols, Total Flavonoids, and Antioxidant Activity

TPC was measured using gallic acid as a reference polyphenol according to a method by Gogna et al. [39]. As such, 0.14 mL of each of UCS or BCS and 0.7 mL of 0.2 N Folin–Ciocalteu were mixed and reacted at room temperature for 8 min. Then, 0.56 mL of 7.5% Na_2_CO_3_ was added and further reacted for 60 min. Absorption of the mixture was measured at 765 nm using a spectrophotometer (UV-1650PC, Shimadzu, Kyoto, Japan) and TPC was expressed in mg gallic acid equivalent (GAE)/g dry matter (DM).

TFC of UCS and BCS was measured using the method described by Chang et al. [40]. As such, 0.1 mL of each UCS or BCS was mixed with 0.3 mL of 95% ethanol and 0.56 mL of distilled water. Then, 1 M potassium acetate and 10% aluminum chloride were added. After 30 min of reaction at room temperature, absorption was measured at 415 nm using a spectrophotometer. Quercetin was used as a reference chemical and the TFC concentration was expressed in mg quercetin equivalent (QE)/g DM.

RSA was measured by a modified method based on Dietz et al. [41]. For this, 0.1 M DPPH was prepared by mixing methanol and the 1.25 mL of solution was mixed with 0.25 mL of UCS or BCS. The mixture was reacted at room temperature for 30 min in the dark condition and the absorption was measured at 517 nm using a spectrophotometer. Distilled water and ascorbic acid were used as a negative and positive control, respectively, and RSA was calculated using the following equation.

(1) $$\mathrm{RSA}\;\left(\%\right)=\left\{1\;-\;\frac{\mathrm{Abs}\;(\mathrm{sample})}{\mathrm{Abs}\;(\mathrm{control})}\right\}\;\times \;100$$

### 3.6. Measurement of Tyrosinase Activity Inhibition and UV-A Absorption

TAI was assessed using the method by Kim et al. [42]. Reaction mixture consisted of 0.2 mL UCS or BCS, 200 µL potassium phosphate buffer (pH 6.8), 200 µL L-DOPA, and 200 µL tyrosinase. After 30 min of enzymatic reaction at 25 °C, absorption was measured by spectrophotometer at 405 nm and the TAI was expressed as a percentage based on control.

(2) $$\mathrm{TAI}\;\left(\%\right)=\left\{1\;-\;\frac{\mathrm{Abs}\;\left(\mathrm{control}\right)\;-\;\mathrm{Abs}\;(\mathrm{sample})}{\mathrm{Abs}\;(\mathrm{control})}\right\}\;\times \;100$$

To compare UV-A absorption of UCS and BCS, each sample was diluted 50-times and absorption was measured at 365 nm using a spectrophotometer. As a control group, absorption was measured by adding disodium phenyl dibenzimidazole (DPD, 0.1 mg/mL), a water-soluble organic component for blocking UV-A that is added to a commercial sunscreen. The UV-A absorption was expressed as a percent as shown in the following equation.

(3) $$\mathrm{UV}-A\;\mathrm{Absorption}\;\left(\%\right)=\left\{1\;-\;\frac{\mathrm{Abs}\;\left(\mathrm{sample}\right)}{\mathrm{Abs}\;\left(\mathrm{control}\right)}\right\}\;\times \;100$$

### 3.7. Cell Viability Assay

Human dermal fibroblasts (HDF) and mouse melanoma (B16F0) cells were obtained from the Cell Application (San Diego, CA, USA) and the Korean Collection for Type Culture (KCTC, Jeongeup, Korea), respectively. The cells were inoculated in DMEM with 10% FBS and 1% penicillin/streptomycin and cultured in a CO_2_ incubator (MCO-18AIC, Sanyo, Osaka, Japan) at 37 °C with 5.0% CO_2_. For the cell viability assay, cells were seeded in a 96-well plate with 1 × 10^4^ cells/well of inoculum density and different concentrations (0.0–0.25 mg/mL) of BCS were treated. After 24 h cultivation, a total of 0.1 mL MTT solution was added to induce formazan formation and 0.1 mL DMSO was added after 3 h to dissolve formazan. Then absorption was measured at 450 nm with a microplate reader (AMR-100, Allsheng, Seoul, Korea) and the cell viability was calculated using the following equation.

(4) $$\mathrm{Cell}\;\mathrm{viability}\;\left(\%\right)=\left\{1\;-\;\frac{\mathrm{Abs}\;(\mathrm{sample})}{\mathrm{Abs}\;(\mathrm{control})}\right\}\;\times \;100$$

### 3.8. Measurement of Gene Expression

Total RNA from B16F0 cell was extracted using AccuPrep^®^ universal RNA extraction kit and the expression levels were measured by spectrophotometer (NanoDrop™ 2000, Thermo Fisher, Waltham, MA, USA). Reverse transcription was performed using the amfiRivert cDNA synthesis platinum master mix (GenDEPOT, Barker, TX, USA). The initial denaturation was performed at 95 °C for 30 s, and the denaturation temperature was 59 °C for 30 s, annealing was at 72 °C for 30 s, and extension was at 72 °C for 7 min; samples were amplified for 35 cycles. The sequences of both forward and reverse primers and the size of the amplified genes are listed in Table 1. The mRNA expression levels of *TRP-2*, *TRP-1*, and *β-actin* were measured using Gel Doc™ XR+ system and Quantity One software (Bio-Rad, Hercules, CA, USA).

### 3.9. Measurement of Main Components

Quantitative and qualitative analysis of polyphenol in UCS and BCS were carried out using HPLC (Agilent 1260, Agilent Tech., Santa Clara, CA, USA) equipped with diode array detector (DAD) and Poroshell 120 EC-C18 column (4.6 × 150 mm, Agilent Technology, Santa Clara, CA, USA). The mobile phase was composed of two solvents: 99.9% acetonitrile (A) and 1.0% acetic acid (B), and gradient elution for the separation of polyphenols was as follows: 0–5 min, 0.0–15.0% A; 5–50 min, 15–50% A; 50–60 min, 50–100% A; and 60–64 min, 100–0.0% A. Main components in UCS and BCS were identified by comparing RT as well as the DAD spectrum (190–640 nm) with polyphenol standards. Phenolic standards including gallic acid, coumaric acid, quercetin, syringic acid, caffeic acid, epigallocatechin gallate, vanillin, ferulic acid, ascorbic acid, vanillic acid, eugenol, chatechin, benzoic acid, chlorogenic acid, and sinapic acid with purity of 99.8% or higher were purchased from Sigma-Aldrich.

The reidentification of the main components detected through HPLC was performed using liquid chromatography-tandem mass spectrometry (LC-MS/MS) with Roc™ C18 column (3.0 × 150 mm, Restek, Bellefonte, PA, USA). The column temperature and injection volume were set at 30 °C and 10 µL. The main components in BCS were analyzed by negative and positive ion electrospray ionization using an electrospray ionization source and mass spectra were operated in the negative and positive ion mode between 100 and 800 *m/z* by triple-quadrupole mass spectrometer (TSQ Quantum Ultra EMR., Thermo Fisher, Waltham, MA, USA). The mobile phase consisted of two solvents: 0.1% formic acid in distilled water (A) and 0.1% formic acid in 50.0% acetonitrile (B), and a gradient elution system for the separation of polyphenols was as follows; 0–11 min, 95.0–0.0% A; 11–14 min, 0.0–0.0% A; 14–15 min, 0.0–95.0% A; and 15–20 min, 95.0–95.0% A.

### 3.10. Statistical Analysis

Data were expressed as the mean ± standard deviation (SD) for all experiments and probabilities (*p*) of chance difference between groups were calculated according to Student’s *t*-test. A statistically significant test means that the test hypothesis is false or should be rejected and the criteria were set at *p* < 0.05.

This section may be divided by subheadings. It should provide a concise and precise description of the experimental results, their interpretation, as well as the experimental conclusions that can be drawn.

## 4. Conclusions

The CS, by-products of chestnut processing, was bioconverted utilizing *L. plantarum* SM4 isolated from kimchi to improve the production of value-added components for cosmetics products with a skin-whitening effect. Furthermore, in order to assess the bioactive functionality of BCS, RSA, TPC, and TFC, markers of secondary metabolite production and antioxidant effects were measured. Comparative investigations revealed that RSA, TPC, and TFC rose by 1.3-, 2.6-, and 5.4-times in BCS compared to UCS, respectively, implying that bioconversion improved the production of bioactive components, thereby simultaneously increasing the production of secondary metabolites and antioxidant activity.

When the skin-whitening effect of BCS was confirmed by measuring the UV-A absorption rates and TAI, they increased by 1.2- and 1.2-times, respectively, which proved statistically significant at *p* < 0.05. BCS treatment has a skin-whitening effect along with UV-A absorption, confirming it has the dual effect of preventing and curing skin troubles caused by UV radiation. In addition, the evaluation of the melanin synthesis mechanism for the verification of BCS’s skin-whitening effect revealed that mRNA expression of *TRP-1* and *TRP-2* significantly decreased as the concentration of BCS increased, confirming the suppression of the activity of key enzymes in melanin synthesis.

In LC-MS/MS analyses of BCS, gallic acid, coumarin, cinnamic acid, quercetin, and hesperidin, known as secondary metabolites of plants, were identified as candidates for major components with the potential for skin-whitening or UV absorption. In the analysis of BCS using HPLC, gallic acid was present in the highest amount among five components, including gallic acid, coumarin, cinnamic acid, quercetin, and hesperidin, identified in the LC-MS/MS analysis. Thus, gallic acid was thought to be responsible for the bioactive properties of BCS, such as skin-whitening and UV-A absorption.

During the bioconversion process, polymers, such as cellulose, polyphenols, polysaccharides, and proteins, were hydrolyzed into small molecules or aglycon by β-glucosidases and other enzymes, increasing the functional and biological activity of BCS. Therefore, our findings suggest that bioconversion of CS using *L. plantarum* SM4 can enhance antioxidant activity and UV-A absorption by enzymatic hydrolysis of macromoleculs in plant cell wall, allowing the development of natural antioxidants and skin-whitening agents with anti-wrinkle benefits [43].

## Figures and Tables

**Table 1:** List of primers used to determine gene expression of *TRP-2* and *TRP-1* using RT-PCR. The sequences of primers for each gene are shown as forward and reverse.

Genes	Forward Primers (5′-3′)	Reverse Primers (5′-3′)	Size (bp)
^1^ *TRP-1*	GCTGCAGGAGCC TTCTTTCTC	AAGACGCTGCACTGCTGGTCT	398
^2^ *TRP-2*	AGAAGTTTGACAGCCCTCC	CTAACCGCAGAGCAACTTG	360
*β-actin*	ACTACCTCATGAAGATCCTG	TTGCTGATCCACATCTGCTG	731

^1^*TRP-1*: tyrosinase-related protein-1; ^2^*TRP-2*: tyrosinase-related protein-2.

## Data Availability

The data will be available upon suitable request.

## Sample Availability

Samples of the compounds are available from the authors.
